# Prevalence of metabolic syndrome among patients with Schizophrenia in Palestine

**DOI:** 10.1186/1471-244X-12-235

**Published:** 2012-12-27

**Authors:** Waleed M Sweileh, Sa’ed H Zyoud, Salah A Dalal, Sami Ibwini, Ansam F Sawalha, Iyad Ali

**Affiliations:** 1 College of Medicine and Health Sciences, Division of Pharmacy An-Najah National University, Nablus, Palestine

**Keywords:** Schizophrenia, Metabolic syndrome, Palestine

## Abstract

**Background:**

Metabolic syndrome (MS) is a cluster of the most dangerous cardiac risk factors and is associated with high mortality. Ethnic differences in metabolic syndrome (MS) criteria and prevalence rates have been reported. The purpose of this study was to investigate the MS prevalence among patients with schizophrenia in Palestine.

**Methods:**

We recruited 250 patients with schizophrenia from 4 psychiatric primary healthcare centers in Northern Palestine. The MS prevalence was assessed based on National Cholesterol Education Program Adult Treatment Panel III Adapted criteria.

**Results:**

The overall MS prevalence was 43.6%, with 39% in male and 55.9% in female patients. On average, the study patients had 2.3 ± 1.3 metabolic abnormalities. Univariate analysis showed that MS was significantly higher with older age, female gender, longer duration of the illness, smoking, abdominal obesity, high systolic and diastolic blood pressure, high triglycerides, low HDL-C, and high fasting plasma glucose. Multiple logistic regression analysis showed that only systolic blood pressure, high triglycerides, high fasting plasma glucose, and low HDL-C were significant predictors of MS in schizophrenic patients.

**Conclusions:**

MS is common among Arab patients with schizophrenia. Patients with schizophrenia should receive regular monitoring and adequate treatment of cardio-metabolic risk factors.

## Background

Schizophrenia is a mental disorder that affects approximately one percent of the various populations throughout the world [1-3]. Patients with schizophrenia are reported to have shorter life span compared to the general population [4-6]. The most commonly reported cause of excess mortality among patients with schizophrenia is cardiovascular diseases [7,8]. Cardiovascular morbidity and mortality among patients with schizophrenia has been linked to high prevalence of metabolic syndrome [9]. Metabolic syndrome (MS), also known as syndrome X or insulin resistance syndrome is a cluster of interrelated metabolic risk factors that appear to directly promote the development of atherosclerotic cardiovascular disease [10-13]. Furthermore, metabolic abnormalities not only have an impact on physical health but also on a poorer quality of life [14], non-compliance [15] and a lower functional outcome [16].

Three definitions of MS have been proposed by the National Cholesterol Education Program Adult Treatment Panel III (NCEP ATPIII), the World Health Organization (WHO) and the International Diabetes Federation (IDF) [17-19]. Regardless of the criteria, five factors, are thought to comprise MS—large waist circumference (WC; as indicator of central obesity), elevated triglycerides (TG), low high-density lipoprotein cholesterol (HDL-C) concentration, high blood pressure and elevated fasting plasma glucose. The most commonly used definitions for the MS are the NCEP ATPIII, and the adapted ATP-III A proposed by the American Heart Association (AHA) following the ADA lowering of the threshold for impaired fasting glucose to 100 mg/dl [20,21]. The IDF stressed the importance of waist circumference, using both more stringent and ethnic-/race-specific criteria in the definition of MS. Recently, IDF and American Heart Association (AHA) and the National Heart, Lung, and Blood Institute (NHLBI) representatives held discussions to attempt to resolve the remaining differences between definitions of metabolic syndrome. Both sides agreed that abdominal obesity should not be a prerequisite for diagnosis but that it is 1 of 5 criteria, so that the presence of any 3 of 5 risk factors constitutes a diagnosis of metabolic syndrome [22].

Recent research on MS in the general population has indeed provided evidence for the development of ethnic-/race-specific criteria [23-25]. Until now, it has remained unclear to what extent these differences in the general population are due to genetic factors or cultural factors such as life style or economic factors. As the criteria of MS as well as life style and economic factors are different between ethnic groups, evaluation of the prevalence of MS in patients with schizophrenia in different ethnic groups is needed.

Many reports have been published on the prevalence of the MS in the general population among Arabs in the Middle East [26-28]. However, reports about the prevalence of MS among Arab patients with schizophrenia are very few. To further investigate the prevalence of metabolic syndrome among Arab patients with schizophrenia, this study was carried out among a sample of patients with schizophrenia in Palestine.

## Methods

### Research design and methods

This is a cross sectional study conducted from August 2011 until February 2012 at governmental primary healthcare psychiatric centers in Northern West-Bank, Palestine. All patients attending the psychiatric healthcare centers during the study period with the following criteria were invited to participate: 1) their age was above 16 years old, and 2) they were diagnosed with schizophrenia as defined by DSM-IV. In order to estimate with sufficient precision the prevalence of MS, we hypothesized that the prevalence of MS to be 30%. We calculated the sample size with a 99% confidence interval and of a 10% width. Based on this, we needed a sample size at least 100 patients.

On the assessment day, we collected demographic and clinical information, including current antipsychotic use, from medical documents of the institutions. The following types of information were collected: gender, age, occupation, marital status, length of psychiatric illness, antipsychotic medications; waist circumference (WC), fasting plasma glucose (FPG) measured in mg/dL, and lipid profile. Focus group discussions were continuously held between the research team to maintain rational of the data collection process. Regular evaluations took place throughout the abstraction period to identify any problems in data collection, the interpretation of definitions, and the application of study criteria. Before commencing data analysis, an extensive series of checks were performed for data consistency, proper sequences of data, and an evaluation of missing or incomplete data. The data collection form was modified by the principal researchers and the modified version was reviewed by experts to ensure content and construct validity. Data from the pre-test evaluation were not included in the final analysis. Approval to perform the study was obtained from the Palestinian ministry of health and the college of Graduate Studies at An-Najah national University and Institutional Review Board (IRB). Written consent was obtained from patients.

The antipsychotic use patterns were recorded by the types and number of administered antipsychotic drugs. As clozapine and olanzapine have the highest potential to cause metabolic abnormalities [29,30] and weight gain [31], we classified the types of antipsychotic drugs into a clozapine/ olanzapine group and other antipsychotics group. The number of antipsychotics used was categorized as monotherapy, currently using only one kind of antipsychotic drug, and combination, currently using more than one kind of antipsychotic. Medical information regarding the duration of current antipsychotic regimen or previous antipsychotic usage were not obtained due to limited related information in their documents. Waist circumference (WC) was measured using an outstretched tape meter, without any pressure to the body surface, and was recorded to the nearest 0.1 cm. The waist measurement was taken with the tape meter in a horizontal plane, midway between the inferior margin of the ribs and the superior border of the iliac crest. To avoid inter-subjective error, all measurements were taken by the same person. Sitting blood pressure (BP) was performed. Two BP measurements were made by two nurses in the right arm after the participant sat and rested for at least 5 min. In case of difference in BP reading, a third one was carried out and the average was calculated. For FPG blood samples were collected from all subjects between 8:00 and 9:00 (a.m.) after 12 h overnight fast. Blood was collected from an ante-cubital vein punctures and was collected while subject or client in a sitting position. FPG was determined using Chemistry kits bought from Human, Germany. Blood samples were taken in a sitting position according to the standard protocol and centrifuged within 30 to 45 minutes of collection. Biochemical analysis was conducted on fasting plasma samples; all blood analyses being done at the University laboratory on the day of blood collection. For lipid measurement, LDL-C, HDL-C, TC, and TG were measured using HUMAN kit, Germany. The MS was defined according to ATPIII definition.

Chlorpromazine equivalents (CPZeq): Total CPZeq was constructed by calculating a total daily dose of each antipsychotic listed in the medical file. Then each converted antipsychotic-specific CPZeq amount is added to arrive at a total dose. The daily dose of antipsychotic medication prescribed to each patient was converted to milligram equivalents of chlorpromazine according to conversion factors derived from the literature [32-34].

The operational definition of antipsychotic combination is the use of two or more antipsychotic drugs while that of monotherapy is the use of one antipsychotic drug.

### Statistical analysis

Descriptive statistics for all study variables were computed. These descriptive statistics included frequencies and percentages for all categorical variables in addition to means, standard deviations and ranges for all normally distributed continuous variables while median and inter quartile range was used for continuous variables that were not normally distributed. Differences between groups were carried out using independent sample *t* test for continuous variable while Chi-square test was used for categorical variables. The size effect for continuous variables was expressed as Cohen’s *d* value using the on line calculator (http://www.uccs.edu/~lbecker/). For categorical variables, the size effect was calculated as OR and then converted to Cohen’s d value based on mathematical equation present in literature [35]. Univariate analysis was carried out using the Binary logistic regression while multiple logistic regression was carried out on variables that showed significance in univariate analysis. In both univariate and multiple logistic regression, the presence of MS was used as the dependent categorical variable. All statistical analyses were conducted using Statistical Package for Social Sciences SPSS (PASW version 18.0; IBM, Somers, NY) statistical packages for Windows. The conventional 5 percent significance level was used throughout the study.

## Results

### General descriptive data

A total of 250 patients diagnosed with schizophrenia were recruited for this study. Mean age of the patients was 41.9 ± 11.8 [95% CI: 40.5 – 43.4] years. No significant difference in age was found between male and female patients (40.3 ± 12.4 for females versus 42.5 ± 11.5 years for males; p = 0.2). The median duration of the psychiatric illness was 15 (Q1 – Q3: 9 – 20) years. The majority (194, 77.6%) of the patients were using first-generation antipsychotic (FGA) while 33 (13.2%) were using second-generation antipsychotics (SGA) and 23 (9.2%) were using both FGA and SGA. Table 1 shows the general characteristics of the sample stratified with gender.

**Table 1:** General characteristics of the study sample

**Variable**	***N *(%) or Mean ± SD**			**P value d (Cohen’s value) and/or OR (95CI)**	**Chi- Square value (*X***^**2**^**) Or *t*-test value**
	**Total**	**Male**	**Female**		
	**250 (100%)**	**182 (73.8%)**	**68 (27.2%)**		
**Age (years)**	41.9 ± 11.8	42.5 ± 11.5	40.3 ± 12.4	0.2^a^	
				d = 0.18	−1.3
**Education**				0.4^b^	
≤ School education	213 (85.2%)	153 (84.1)	60 (88.2)	1.4 (0.62 – 3.3)	
College education	37 (14.8%)	29 (15.9)	8 (11.8)	d = 0.18	0.68
**Marital Status**				<0.01 ^b^	
Married	138 (55.2%)	88 (48.4)	50 (73.5)	3 (1.6 – 5.5)	
Single/Divorced	112 (44.8%)	94 (51.6)	18 (26.5)	d = 0.61	12.7
**Smoker**				< 0.01 ^b^	
Yes	153 (61.2%)	55 (80.9)	42 (23.1)	14.1 (7 – 28.3)	
No	97 (38.8%)	13 (19.1)	140 (76.9)	d = 1.46	69.66
**Occupation**				0.1 ^b^	
Not working	219 (87.6%)	156 (85.7)	63 (92.6)	2.1 (0.8 – 5.7)	
Working	31 (12.4%)	26 (14.3)	5 (7.4)	d = 0.41	2.2
**Duration of psychiatric illness**	15.8 ± 9.3	16.2 ± 9.0	14.8 ± 9.9	0.3 ^a^	
				d = 0.15	−1.01
**Waist Circumference**				< 0.01 ^b^	
Normal	137 (54.4)	124 (68.1%)	12 (17.6%)	0.1 (0.05 – 0.2)	
Above Normal	114 (45.6%)	58 (31.9%)	56 (82.4%)	d = −1.27	50.86
**Systolic Blood Pressure**	124.2 ±14.4	125.1 ± 14.1	121.8 ± 15	0.1 ^a^	−1.6
				d = 0.23	
**Diastolic Blood Pressure**	75.5 ± 10.8	76.4 ± 10.2	73 ± 12.1	0.04 ^a^	−2.0
				d = 0.3	
**Triglycerides**	193.2 ± 180.2	187.3 ± 125.4	208.8 ± 279	0.5 ^a^	0.6
				d = −0.1	
**HDL-C**	44.9 ± 10.9	43.8 ± 11	46.4 ± 10.6	0.1 ^a^	1.7
				d = −0.24	
**FPG**	99.5 ± 47.5	96.6 ± 45.3	107.3 ± 52.6	0.1 ^a^	1.5
				d = −0.2	
**CPZeq**	436.9 ± 275.6	455.6 ± 283.7	386.8 ± 247.7	0.07 ^a^	−1.9
				d = 0.3	
**Olanzapine/Clozapine**				0.9 ^b^	
Yes	30 (12)	22 (12.1)	8 (11.8)	1.2 (0.6 – 2.3)	0.18
No	220 (88)	160 (87.9)	60 (88.2)	d = 0.1	
**Drug regimen**				0.1 ^b^	
Monotherapy	124 (49.6)	85 (46.7)	39 (57.4)	1.5 (0.9 – 2.7)	2.2
Combination therapy	126 (50.4)	97 (53.3)	29 (42.6)	d = 0.22	

Abbreviations: *SD*, Standard deviation; *OR*, Odds ratio; *CI*, confidence interval**;***HDL-C*, High-density lipoprotein cholesterol; *FPG*, Fasting plasma glucose; *CPZeq*, Chlorpromazine equivalents.

^a^ Statistical significance of differences estimated with the independent Student’s *t* test.

^b^ Statistical significance of differences estimated with the Chi square test.

The most common antipsychotic medication used by the patients was chloropromazine tablet (128; 31.5%), followed by fluphenazine IM depot injection (125; 30.8%), haloperidol tablet (74; 18.2%), clozapine (35, 8.6%), olanzapine (15, 3.7%), haloperidol decanoate (11, 2.7%), risperidone (8, 2%), trifluperazine (7, 1.7%), thioridazine (1, 0.2%) and zuclopenthixol (2, 0.5%). Table 1 shows the demographic and clinical characteristics of the participants.

### Prevalence of metabolic syndrome

According to the ATP-III A criteria: 109 (43.6%) patients met the criteria for the syndrome (3 or more MS criteria) while141 (56.4%) patients did not meet the full criteria for the syndrome (2 or less MS criteria); (Table 2). On average, the study patients had 2.3 ± 1.3 metabolic abnormalities. Table 3 displays the distribution of metabolic components as defined by ATP-III A criteria among the recruited patients. Among males, high TG was the most common metabolic component while abdominal obesity was the most common among females (Table 4). High FPG was the least common metabolic dysregulation in both genders.

**Table 2:** Prevalence of metabolic abnormalities using ATP-III a criteria among 250 patients with schizophrenia in Northern Palestine

**Number of MS criteria**	**N (%)**	**Gender**	
		**Male**	**Female**
0	22 (8.8%)	21(11.5%)	1 (1.5%)
1	56 (22.4%)	46 (25.3%)	10 (14.7%)
2	63 (25.2%)	44 (24.2%)	19 (27.9%)
3	52 (20.8%)	38 (20.9%)	14 (20.6%)
4	47 (18.8%)	28 (15.4%)	19 (27.9%)
5	10 (4%)	5 (2.7%)	5 (7.4%)
MS ≥ 3	109 (43.6%)	71 (39%)	38 (55.9%)

**Table 3:** Univariate and logistic regression analysis of metabolic syndrome of 250 patients

**Variable**	**With MS *N *(%) or Mean ± SD**	**Without MS *N *(%) or Mean ± SD**	**Unadjusted OR (95% CI)**	**Adjusted OR (95% CI)**
**Age** (years)	45.1 ± 10.4	39.5 ± 12.2	1.04 (1.02 –1.07)	0.98 (0.93 – 1.03)
**Gender**				
Female	38 (55.9%)	30 (44.1%)	2 (1.1 – 3.5)	1.91 (0.64 – 5.69)
Male	71 (39%)	111 (61%)		
**Duration**				
< 10 years	90 (42.3%)	123 (57.7%)	2.2 (1.3 – 3.8)	1.94 (0.62 – 6.01)
>10 years	19 (51.4)	18 (48.6%)		
**Smoking**				
Yes	55 (35.1%)	98 (64.9%)	2.2 (1.3 – 3.8)	2.16 (0.82 – 5.69)
No	54 (55.7%)	43 (44.3%)		
**Marital status**				
Married	53 (47.3%)	59 (52.7%)	0.76 (0.46 – 1.25)	–
Single	56 (40.6%)	82 (59.4%)		
**Education**				
< 12 years	90 (42.3%)	123 (57.7%)	1.4 (0.7 – 2.9)	–
>12 years	19 (51.4%)	18 (48.6%)		
**Occupation**				
Employed	12 (38.7%)	19 (61.3%)	0.8 (0.36 – 1.7)	–
Unemployed	97 (44.3%)	122 (55.7%)		
**Family history of DM**				
Yes	52 (46.4%)	60 (53.6%)	1.2(0.75 – 2.04)	–
No	57 (41.3%)	81 (57.7%)		
**CPZeq** (mg)	432 ± 284.3	440.6 ± 269.7	1(0.9 – 1)	–
**Therapeutic Regimen**				
Monotherapy	53 (42.7%)	71 (57.3%)	1.07(0.65 – 1.8)	–
Combination	56 (44.4%)	70 (55.6%)		
**Depot antipsychotic**				
Yes	68 (49.3)	70 (50.7%)	1.7 (1.01 – 2.8)	1.74 (0.73 – 4.14)
No	41 (36.6%)	71 (63.4%)		
**Olanzpine/Clozapine**				
Yes	10 (33.3%)	20 (66.7%)	0.61 (0.3 – 1.4)	–
No	99 (45%)	121 (55%)		
**WC** (cm)	105.1 ± 13.2	92.3 ± 10.7	1.1 (1.07 – 1.1)	1.09 (1.05 – 1.13)
**Systolic BP** (mmHg)	131.1 ± 12.9	118.9 ± 13.2	1.07 (1.05 – 1.1)	1.11 (1.07 – 1.16)
**Diastolic BP** (mmHg)	78.4 ± 10.1	73.2 ± 10.9	1.05 (1.02 – 1.07)	0.96 (0.92 – 1.01)
**TG** (mg/dl)	256.4 ± 224.6	144.3 ± 115.4	1 (1 – 1)	1.01 (1.0 – 1.01)
**HDL** (mg/dl)	42.4 ± 11.8	46.2 ± 9.9	0.96 (0.94 – 0.99)	0.95 (0.91 – 1.00)
**LDL** (mg/dl)	121.7 ± 36.8	112.1 ± 39.1	1 (1 – 1.01)	
**FPG** (mg/dl)	120.5 ± 64.5	83.3 ± 13.9	1.05 (1.03 – 1.07)	1.06 (1.03 - 1.09)

Abbreviations: *SD*, Standard deviation; *OR*, Odds ratio; *CI*, confidence interval; *MS*, Metabolic syndrome; *DM*, diabetes mellitus; *WC*, Waist Circumference; *HDL*, High-density lipoprotein; *LDL*, low-density lipoprotein; *BP*, Blood pressure; *TG*, triglyceride; *FPG*, Fasting plasma glucose; *CPZeq*, Chlorpromazine equivalents.

**Table 4:** Prevalence of metabolic dysregulations in patients with schizophrenia

**MS ATP-III A**	**All Frequency (%)**	**Male**	**Female**	**OR (95% CI) and d (Cohen’s value)**	**P**	**Chi-Square value**
**WC** (M > 102 cm; F > 88 cm)	114 (45.6%)	58 (31.9%)	56 (82.4%)	0.1 (0.05 – 0.2) d = −1.3	<0.01	50.9
**FPG** (≥100 mg/dL)	80 (32%)	52 (28.6%)	28 (41.2%)	0.6 (0.3 – 1.02) d = −0.28	0.06	3.6
**HDL** (M < 40 mg/dl; F < 50 mg/dl)	130 (52%)	87 (47.8%)	43 (63.2%)	0.53 (0.3 – 0.94) d = −0.35	0.03	4.7
**BP** (≥ 130/85 mmHg)	129 (51.6%)	92 (50.5%)	31 (45.6%)	1.2 (0.68 – 2.1) d = 0.1	0.55	0.35
**TG** (> 150 mg/dl)	123 (49.2%)	96 (52.7%)	33 (48.5%)	1.2 (0.7 – 2.1) d = 0.1	0.5	0.49

Abbreviations: *MS*, Metabolic syndrome; *ATP-III*, Adult Treatment Panel III; *OR*, Odds ratio; *CI*, confidence interval; *WC*, Waist Circumference; *M*, Male; *F*, Female; *HDL*, High-density lipoprotein; *FPG*, Fasting plasma glucose; *BP*, Blood pressure; *TG*, triglyceride.

The frequency of abdominal obesity (WC) and low HDL-C in female patients was significantly higher than that in males. The overall prevalence of MS was 43.6%. Female patients had higher MS prevalence than male patients.

### Univariate analysis and logistic regression

Univariate analysis (Table 3) shows that cases with MS were significantly older, being females, having longer duration of the illness, smokers, had higher systolic and diastolic BP, higher waist circumference, higher TG, FPG, and lower HDL compared to cases without MS. Borderline significance was found between cases with and without MS with regard to using depot antipsychotic medications and LDL level. On the other hand there was no significant difference between both groups with regard to other variables. Multiple logistic regression (Table 3) which included significant variables in univariate analysis indicated that SBP, TG, FPG, HDL and WC were significantly associated with MS cases.

## Discussion

In this study we investigated prevalence of MS among a sample of Arab patients with schizophrenia. We estimated that approximately 44% of the study sample has the metabolic syndrome. The high prevalence of the metabolic syndrome in the present study has several clinical implications. First, there is a crucial need to develop methods, including physical activity and nutrition, to control metabolic abnormalities among schizophrenic patients. Second, education and training are needed to ensure that mental healthcare workers have the knowledge and skills necessary to identify schizophrenic patients with the metabolic syndrome. Third, close collaboration between mental healthcare workers and other physicians is needed to establish better healthcare for patients with schizophrenia. Finally, use of antipsychotics with lesser metabolic side effects is needed in patients at risk of developing MS.

Even though there is a considerable variation in methodology and criteria among published studies, the MS prevalence in our study was within the range of prior reports published regionally and worldwide [36-42]. Studies about prevalence rate of MS obtained in the Arab world are few. A study carried out among 63 patients to determine the risk factors of metabolic syndrome in a sample of Egyptian patients with schizophrenia using the IDF criteria found that twenty four patients (38.09%) met the criteria for MS [42]. Another study carried out among 220 UAE psychiatric inpatients using the NCEP ATP III criteria found that the prevalence of metabolic syndrome was about 48.1% [43].

Our study showed that female patients had higher risk of having MS, which is a common finding in previous studies [36,38-41]. In a between gender comparison, a higher proportion of central obesity and low HDL-C in female patients with schizophrenia in this study was consistent with observations in the literature [38-40].

We did not find a relationship between the presence of MS and types of antipsychotic drug. The relationship between antipsychotic drugs and risk of developing MS has remained divergent in the literature [37,41,44,45]. Our results have found no significant difference in the antipsychotic dose measured as CPZeq or antipsychotic regimen and the presence or absence of MS. A study has reported that those receiving a combination of SGA and FGA had a higher risk of developing MS [46]. Another study showed no difference on MS profiles between patients using FGA, SGA, or combination therapy [47]. Despite the clear evidence that antipsychotic drugs are implicated in metabolic disease in patients with schizophrenia, there is increasing evidence that schizophrenia itself is an independent risk factor [48,49].

It is well known that MS increases the risk of developing cardiovascular disease (CVD) and diabetes. A study has shown that metabolic syndrome accounts for up to one third of CVD in men and approximately half of new Type II diabetes mellitus over 8 years of follow-up [50]. It was suggested that CVD risk factors have an additive effect on risk for CVD, and the same statement is generally true for metabolic syndrome traits and risk for CVD and Type II DM [51]. However, Framingham model remains the most commonly validated risk model for CVD [52].

This study has three limitations. First, we enrolled subjects with stable schizophrenia state while acutely ill and more chronically sedentary patients were not included in this study which affects the generalization of the results. Second, the duration of antipsychotic drug use in this cross-sectional design was not obtained. Switching of antipsychotics is common among psychiatric patients. So the prevalence of MS between types of antipsychotics did not show significant difference. Third, factors that potentially affect the development of MS, such as lifestyle or genetic variations were not investigated.

## Conclusions

In conclusion, this study indicates that the metabolic syndrome is prevalent in Arab patients with schizophrenia. Physicians treating patients with schizophrenia are recommended to monitor the components in the metabolic syndrome to identify those subjects with an increased risk for cardiovascular disorders. In addition, patient education and proper selection of antipsychotic agents are needed.

## Abbreviations

MS: Metabolic syndrome; NCEP ATPIII: National cholesterol education program adult treatment panel III; IDF: International diabetes federation; AHA/NHLBI: American heart association/national heart lung, and blood institute; ADA: American diabetes association; WC: Waist circumference; FPG: Fasting plasma glucose; HDL-C: High density lipoprotein – cholesterol; LDL-C: Low density lipoprotein – cholesterol; TC: Total cholesterol; TG: Triglycerides; BP: Blood pressure; SBP: Systolic blood pressure; DBP: Diastolic blood pressure; SGA: Second generation antipsychotics; CPZeq: Chlorpromazine equivalents; CVD: Cardiovascular disease.

## Competing interest

The authors declare that they have no competing of interests.

## Authors’ contributions

WS: Concept, idea, literature review and manuscript writing. SZ: Statistical Analysis. SD: Data collection. SE: Data collection. AS: Concept and idea. IA: Manuscript writing. All authors read and approved the final manuscript.

## Pre-publication history

The pre-publication history for this paper can be accessed here:

http://www.biomedcentral.com/1471-244X/12/235/prepub
